# Administration of Glutaredoxin-1 Attenuates Liver Fibrosis Caused by Aging and Non-Alcoholic Steatohepatitis

**DOI:** 10.3390/antiox11050867

**Published:** 2022-04-28

**Authors:** Yuko Tsukahara, Beatriz Ferran, Erika T. Minetti, Brian S. H. Chong, Adam C. Gower, Markus M. Bachschmid, Reiko Matsui

**Affiliations:** 1 Vascular Biology Section, Whitaker Cardiovascular Institute, Boston University School of Medicine, Boston, MA 02118, USA; ytsuka@bu.edu (Y.T.); beatriz-ferranperez@ouhsc.edu (B.F.); eminetti@bu.edu (E.T.M.); chongbsh@bu.edu (B.S.H.C.); markus@quintarabio.com (M.M.B.); 2 Clinical and Translational Science Institute, Boston University School of Medicine, Boston, MA 02118, USA; ctsibio@bu.edu

**Keywords:** liver fibrosis, aging, non-alcoholic steatohepatitis, glutaredoxin-1, immune cells

## Abstract

Liver fibrosis is a sign of non-alcoholic fatty liver disease progression towards steatohepatitis (NASH) and cirrhosis and is accelerated by aging. Glutaredoxin-1 (Glrx) controls redox signaling by reversing protein *S*-glutathionylation, induced by oxidative stress, and its deletion causes fatty liver in mice. Although Glrx regulates various pathways, including metabolism and apoptosis, the impact of Glrx on liver fibrosis has not been studied. Therefore, we evaluated the role of Glrx in liver fibrosis induced by aging or by a high-fat, high-fructose diet. We found that: (1) upregulation of Glrx expression level inhibits age-induced hepatic apoptosis and liver fibrosis. In vitro studies indicate that Glrx regulates Fas-induced apoptosis in hepatocytes; (2) diet-induced NASH leads to reduced expression of Glrx and higher levels of S-glutathionylated proteins in the liver. In the NASH model, hepatocyte-specific adeno-associated virus-mediated Glrx overexpression (AAV-Hep-Glrx) suppresses fibrosis and apoptosis and improves liver function; (3) AAV-Hep-Glrx significantly inhibits transcription of Zbtb16 and negatively regulates immune pathways in the NASH liver. In conclusion, the upregulation of Glrx is a potential therapeutic for the reversal of NASH progression by attenuating inflammatory and fibrotic processes.

## 1. Introduction

Liver fibrosis is a sign of non-alcoholic fatty liver disease (NAFLD) progression towards steatohepatitis and cirrhosis. The higher level of fibrosis in non-alcoholic steatohepatitis (NASH) is associated with a higher risk of developing liver cirrhosis, hepatocellular carcinoma [1,2], and cardiovascular disease [3,4]. Therefore, critical endpoints of NAFLD clinical trials include halting and reversing the progression of hepatic fibrosis [5]. However, there are no FDA-approved treatments for NAFLD or NASH; primary treatment is limited to lifestyle modifications and liver transplantation for advanced cases [5]. Oxidants and lipotoxicity contribute to NAFLD severity and progression to NASH [6,7,8,9,10]. The antioxidant vitamin E shows positive effects in resolving NASH in clinical trials [7,11,12,13], but recent guidelines limit vitamin E as a short-term treatment for adults with biopsy-proven NASH [14].

Aging is also a risk factor for fibrosis progression in chronic liver diseases [15]. Older patients with non-alcoholic fatty liver disease (NAFLD) are more prone to develop non-alcoholic steatohepatitis (NASH) with fibrosis and inflammation than young patients [16,17]. In the context of a high-fat diet, aging also augments fibrosis, rather than steatosis, in the mouse liver [18,19]. In addition, aged mice show exacerbated liver fibrosis induced by carbon tetrachloride in association with an impaired resolution of the extracellular matrix [20,21].

We previously showed that mice with deletion of the glutaredoxin-1 (Glrx) gene develop NAFLD with regular chow and are more susceptible to diet-induced inflammation [22]. Glrx, or thioltransferase, reverses oxidative thiol modification of protein with glutathione (*S*-glutathionylation). Glrx is often categorized as an antioxidant, but its deletion does not necessarily increase sensitivity to oxidative stress [23,24]. Glrx regulates various biological functions, including activation of SirT1 [22,25] and prevention of apoptosis [26]. In addition, upregulation of Glrx attenuates experimental lung fibrosis [27]. However, the effect of Glrx on liver fibrosis and NASH progression is not known.

In this study, we examine the extent to which Glrx can control age-induced liver fibrosis. We found that biological aging *per se* induced mild fibrosis and increased glutathionylated protein levels in the liver of mice fed a regular chow diet. As a proof of concept, we administrated hepatocyte-specific adeno-associated virus (AAV-Hep)-mediated Glrx and showed decreased apoptosis and collagen accumulation in the liver of aged mice. Furthermore, we tested the effects of AAV-Hep-Glrx on high-fat, high-fructose diet-induced NASH in mice and demonstrated that AAV-Hep-Glrx attenuated diet-induced fibrosis and improved hepatic function. Gene expression profiling of the liver indicated that AAV-Hep-Glrx suppressed leukocyte-related inflammatory pathways. In particular, the expression of Zbtb16 (zinc finger and BTB domain-containing 16) was significantly decreased by Glrx administration, suggesting that Zbtb16 may be a key downstream effector controlling liver fibrosis. Interestingly, the diet change to regular chow indicates that AAV-Hep-Glrx has an anti-fibrotic effect independent of steatosis.

In summary, hepatocyte-targeted upregulation of Glrx inhibits liver fibrosis in chow-fed aged mice and in diet-induced NASH, and AAV-Hep-Glrx is a potential therapeutic for the reversal of NASH progression by attenuating inflammatory and fibrotic processes.

## 2. Materials and Methods

### 2.1. Reagents

The following antibodies were purchased; anti-glutaredoxin-1 (Glrx) (Cayman Chemicals, Ann Arbor, MI, USA), anti-cleaved caspase 3 (Cell Signaling Technology, Danvers, MA, USA), anti-α-smooth muscle actin (αSMA) (Abcam, Cambridge, UK), anti-glutathione (Pr-SSG) (Virogen, Watertown, MA, USA), anti-4-hydroxynonenal (HNE) (Abcam), anti-goat IgG-HRP (Santa Cruz, Dallas, TX, USA), anti-rabbit IgG-HRP, and anti-mouse IgG-HRP (Kindle Biosciences, Greenwich, CT, USA). All cell culture reagents were purchased from Gibco (Waltham, MA, USA), Lonza (Portsmouth, NH, USA), and Atlanta Biologicals (Flowery Branch, GA, USA). Chemicals for liver perfusion and AAV production were purchased from Sigma (St. Louis, MO, USA) and Fisher Scientific (Waltham, MA, USA) unless specified otherwise.

### 2.2. Mice

Glrx KO mice were originally generated by Dr. Y.S. Ho (Wayne State University, Detroit, MI, USA) [23], backcrossed to C57BL/6NJ background at Janssen-Heininger’s laboratory at the University of Vermont and imported to Boston University. We further backcrossed the mice (>10 generations) onto C57BL/6J. Male Glrx KO mice and wild-type (WT) littermates were used in the study. Glrx transgenic (TG) mice were generated with human Glrx (hGlrx) transgene driven by the human β-actin promoter at the laboratory of Y.S. Ho (Wayne State University, Detroit, MI, USA) [28]. Heterozygous Glrx TG mice and WT littermates (males and females, 19–22 months old) were used in this study. For the diet-induced NASH model, we fed 10-week-old mice a high-fat high-fructose diet containing 40 Kcal% fat (palm oil), 20 Kcal% fructose, and 2% cholesterol (Opensource diet D09100310, Research Diets) for 32 weeks. The C57BL/6J mice fed this diet developed a NASH phenotype, including steatosis, lobular inflammation, hepatocyte ballooning, and fibrosis. NAFLD activity score (NAS) was reported as about 5–7 [29,30]. A cohort of mice switched from this diet (named as NASH diet) after 28 weeks to a regular chow (4.5 Kcal% fat) for 4 more weeks. Mice were maintained in the Animal Science Center at Boston University Medical Campus, and animal study protocols were approved by the Institutional Animal Care and Use Committee at Boston University (Protocol No. 201800225).

### 2.3. Collagen Staining, Hematoxylin and Eosin (H&E) Staining

Liver samples were fixed in 10% formalin overnight and replaced into 10% and 20% sucrose before embedding in OCT compound. Frozen OCT-embedded samples were cut to 10 µm sections with a Leica cryostat. The specimens on a slide were washed in PBS for 10 min and incubated in Picro Sirius Red solution (Sirius Red F3B in a saturated solution of picric acid) (Poly Scientific R&D Co., Bay Shore, NY, USA) for 1 h. After dehydration by sequential ethanol and xylene immersions, a cover glass was placed using Permount (Fisher, Waltham, MA, USA) mounting medium. Images were taken by Olympus microscope using a polarized filter (U-POT polarizer, Olympus, Tokyo, Japan). Collagen type I signal appears bright orange in the dark background under polarized light. For quantification of the collagen area, we measured 6–7 images of each liver section. The images were split into 3 colors (red, green, and blue) with ImageJ. Red signals were converted to black and white images. The collagen area (%) was quantified by the percent of the white area in the entire field by ImageJ. Each dot in the graph indicates the average of 6–7 images from one mouse. H&E staining of formalin-fixed Chow/NASH-diet liver was conducted with H&E staining kit (Newcomer Supply, Middleton, WI, USA) following the protocol provided by the manufacturer.

### 2.4. Gene Expression

Tissue samples were powdered in liquid nitrogen. RNA was isolated from tissue powders or cells using TRIzol reagent (Invitrogen, Waltham, MA, USA) following the manufacturer’s protocol. Reverse transcription was performed with RNA to generate cDNA using iScript cDNA synthesis kit (BioRad, Hercules, CA, USA). Quantitative PCR was performed with TaqMan assays (Applied Biosystems, Waltham, MA, USA) or validated SYBR Green assays (BioRad) using a CFX Real-Time System (BioRad). The following TaqMan primers were used: human GLRX (Hs00829752_g1) (for detecting the human GLRX gene in Glrx Tg mice), Acta2 (Mm01546133_m1), Tnf (Mm00443258_m1), and 18S (Mm03928990_g1). Primers for SYBR Green assays are listed in Table 1.

### 2.5. Isolation of Hepatocytes and Primary Culture

Preparation of the solution for hepatocyte isolation was performed according to Mederacke et al. [31]. Mice were anesthetized with isoflurane, and the portal vein was exposed through an abdominal incision and cannulated to perfuse the liver. The inferior vena cava was severed to exsanguinate. Sequential perfusion with EGTA, pronase (Sigma, St. Louis, MO, USA), and collagenase type D (Sigma) solution was performed using a peristaltic pump. The excised liver was minced in DMEM and filtered through a 70-μm cell strainer. The suspension was centrifuged at low speed (6 RCF, 10 min, at 25 °C) to collect hepatocytes. Hepatocytes were seeded on 0.1% gelatin-coated plates in DMEM with 10% FBS and 1% antibiotic and antimycotic solution.

### 2.6. Western Blot

Tissue powder or cultured cells were homogenized in lysis buffer containing 50 mM Tris pH 7.4, 150 mM NaCl, 5 mM EDTA, 1% NP-40, and Protease Inhibitor Cocktail (Halt). After centrifugation (16,800 RCF, 15 min, 4 °C), the supernatant was mixed with LDS sample buffer (Invitrogen); 2-mercaptoethanol was added to 2.5% final concentration (except for the detection of *S*-glutathionylated protein (Pr-SSG), which requires non-reducing conditions). In addition, 10 mM N-ethylmaleimide (NEM) was added to alkylate free protein thiol to detect Pr-SSG. Samples were loaded on a NuPAGE 4–12% Bis-Tris gel (Invitrogen) using NuPAGE MES SDS running buffer (Invitrogen) and transferred to a PVDF membrane (Thermo Fisher Scientific, Waltham, MA, USA) using a Trans-Blot^TM^ Turbo Transfer System (Bio-Rad). The membrane was blocked with 3% non-fat dry milk in TBS-T for 1 h (for Pr-SSG detection, 3% BSA with 25 mM NEM was used) and incubated with primary antibodies overnight at 4 °C in 3% BSA. After washing with TBS-T, membranes were incubated for 1 h with the corresponding HRP-conjugated antibodies: anti-goat IgG-HRP (1:5000), Digital anti-rabbit IgG-HRP (1:3000), or Digital anti-mouse IgG-HRP (1:3000). The chemiluminescent signal was detected using the Hi/Lo Digital-ECL^TM^ Western Blot Detection Kit and the KwikQuant Imager. The signals were quantified by ImageJ software and normalized by Ponceau S staining intensity.

### 2.7. Adeno-Associated Viruses (AAV) Production and In Vivo Injection

The AAV plasmid for hepatocyte-specific overexpression pAAV.TBG.PI.Null.bGH [32] was purchased from Addgene (#105536). Mouse Glrx with a HA/FLAG sequence and a woodchuck hepatitis virus post-transcriptional regulatory element (WPRE) were inserted after the promoter of Serpina7 (thyroxine-binding globulin, TBG), resulting in a pAAV-TBG-Glrx plasmid. AAV production and purification methods were described in detail in our publication [33]. Briefly, pAAV-TBG-Glrx or pAAV-TBG-Cont (empty plasmid) was co-transfected with pAAV-DJ Rep-Cap plasmid (VPK-420-DJ, Cell Biolabs, San Diego, CA, USA) and pHelper (Accession #: AF369965, Stratagene, San Diego, CA, USA) in HEK293T cells. AAVs were collected 6 days later from cells and medium by precipitation with polyethylene glycol (PEG) 8000. Precipitated AAV was purified by chloroform extraction and further purified by discontinuous iodixanol gradient ultracentrifugation for in vivo study. The virus titer was measured by qPCR with primer against the WPRE (Forward: 5′-GGCTGTTGGGCACTGACAAT-3′; Reverse: 5′-CCGAAGGGACGTAGCAGAAG-3′). AAV-Cont or AAV-Glrx (1 × 10^12^ vg) was administrated per mouse by intraperitoneal injection. The promoter of TBG induces a hepatocyte-specific gene expression [32,34]. Thus, we termed the final product AAV encoding TBG promoter-driven-Glrx, as AAV-Hep-Glrx.

### 2.8. Liver and Serum Biochemical Measurements

Hepatic triglyceride and cholesterol levels were measured by the Infinity triglycerides and total cholesterol reagent kit (Thermo Fisher). Serum alanine aminotransferase (ALT) level and aspartate aminotransferase (AST) level were measured by Catalyst Dx Chemistry Analyzer (IDEXX) in the Boston University Analytical Instrumentation Core.

### 2.9. Microarray

We examined the transcriptome of the livers of mice NASH model after AAV-Glrx or AAV-control injection by Affymetrix Mouse Gene 2.0 ST GeneChip microarray (Thermo Fisher) at the Microarray and Sequencing Resource at Boston University School of Medicine. RNA was isolated from mouse liver (*n* = 5 or 6 each), pooled into 3 samples per group. The samples were analyzed after confirming the quality of the RNA by 2100 Bioanalyzer (Agilent Technologies, Santa Clara, CA, USA). Raw CEL files were normalized to produce gene-level expression values with the Robust Multiarray Average (RMA) [35] using the affy Bioconductor R package (version 1.62.0, bioconductor.org) and Entrez Gene-specific R packages from the Molecular and Behavioral Neuroscience Institute (Brainarray) at the University of Michigan (version 24.0.0, brainarray.mbni.med.umich.edu) [36]. Differential expression was assessed using the moderated (empirical Bayesian) *t*-test implemented in the limma Bioconductor R package (version 3.39.19, bioconductor.org) (i.e., creating simple linear models with lmFit, followed by empirical Bayesian adjustment with eBayes). Correction for multiple hypothesis testing was accomplished using the Benjamini–Hochberg false discovery rate (FDR). Human homologs of mouse genes were identified using the NCBI HomoloGene resource (version 68, ftp.ncbi.nih.gov/pub/HomoloGene). All microarray analyses were performed using the R environment for statistical computing (version 3.6.0, www.r-project.org). Gene Set Enrichment Analysis (GSEA) (version 2.2.1, Broad Institute, www.gsea-msigdb.org) [37] was used to identify biological terms, pathways, and processes that were coordinately up- or down-regulated with respect to AAV-Hep-Glrx administration relative to AAV-Hep-control. The Entrez Gene identifiers of the human homologs of the genes interrogated by the array were ranked according to the moderated *t* statistic, and any mouse genes with multiple human homologs (or vice versa) were removed prior to ranking thus that the ranked list represents only those human genes that match exactly one mouse gene. This ranked list was then used to perform pre-ranked GSEA analyses (default parameters with random seed 1234) using the Entrez Gene versions of the H (Hallmark), C2 CP (Biocarta, KEGG, PID, Reactome, WikiPathways), C3 (transcription factor and microRNA motif), and C5 (Gene Ontology, GO) gene sets obtained from the Molecular Signatures Database (MSigDB), (version 7.4. Broad Institute, www.gsea-msigdb.org).

### 2.10. Statistical Analysis

For standard statistical analysis, we used GraphPad Prism 9.0.

Student’s unpaired *t*-test was used for normally distributed data. For the analysis of more than two groups, one-way or two-way ANOVA was used, followed by Bonferroni post-hoc tests. *p* < 0.05 values were considered significant.

## 3. Results

### 3.1. Glrx Reduces Age-Induced Fibrosis in the Liver

**Figure 1:**
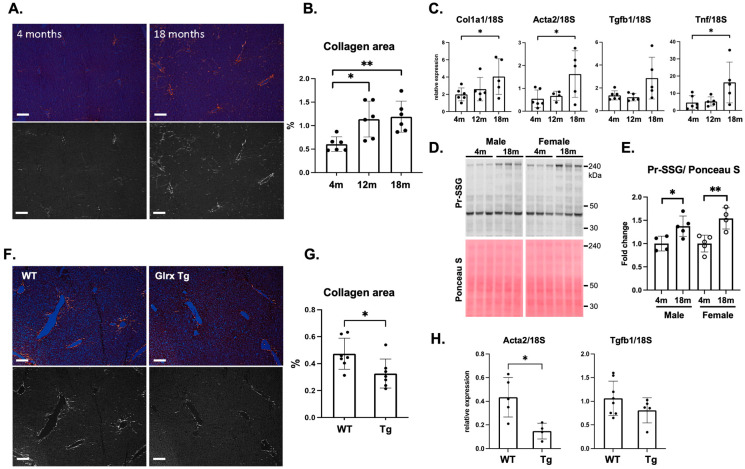
Glrx overexpression inhibits age-induced liver fibrosis in chow-fed mice. (**A**)**.** Picro Sirius Red staining in the liver of 4- and 18-month-old mice. The original image was taken by a microscope with a polarized filter, with a bright red signal indicating collagen (upper photos). The red channel was converted to grayscale to measure the collagen area by ImageJ (lower photos). Scale bar: 50 um. (**B**)**.** Quantitative assessment of collagen area (%) (*n* = 6 mice each age, males, and females). (**C**). Real-time PCR for fibrosis marker genes in the liver (*n* = 5–6, males, and females). Col1a1: collagen type 1, alpha 1; Acta2: alpha-actin 2; Tgfb1: transforming growth factor-beta 1; Tnf: tumor necrosis factor. Relative quantity normalized to 18S is shown. (**D**)**.** Total *S*-glutathionylated protein level (Pr-SSG) in the mouse liver at 4 and 18 months old. (**E**)**.** Semi-quantitative analysis of the blots of Pr-SSG normalized by Ponceau S staining is shown in the graph (*n* = 4). (**F**)**.** Representative images of Picro Sirius Red staining in aged wild type (WT) and Glrx transgenic (TG) mouse liver. The original (upper) and grayscale extracted red channel (lower). Scale bar: 50 um. (**G**)**.** Quantitative assessment of collagen accumulated area (%) (*n* = 7, males and females, 19–22 months old). (**H**)**.** Real-time PCR analysis for fibrosis genes in aged WT and Glrx TG liver. * *p* < 0.05, ** *p* < 0.01. Error bars indicate standard error.

To examine the extent to which biological aging increases fibrosis, we measured collagen deposition by Picro Sirius Red staining in the livers of 4, 12, and 18-month-old male and female C57BL/6J mice (National Institute of Aging depository). The quantitative assessment of collagen-stained areas indicated an increase in collagen deposition in the livers of 12- and 18-month-old mice compared to 4-month-old mice (Figure 1A,B). In addition, the expression of fibrotic markers (Col1α1, αSMA/Acta2, Tgfb1) and Tnf were increased in the livers of 18-month-old mice (Figure 1C), suggesting that fibrotic processes and inflammation were promoted without exogenous stimulus in the aged liver. Total S-glutathionylated protein (Pr-SSG) was also measured in the liver by Western blot. In both male and female mice, the total Pr-SSG level was increased by aging, which is indicative of enhanced oxidative stress in aged liver (Figure 1D,E). Increased oxidants may cause hepatocyte damage and liver inflammation, leading to collagen accumulation in the aged liver. Furthermore, we examined liver fibrosis in aged hGlrx transgenic (TG) mice that constitutively express human Glrx under the β-actin promotor [28]. In Glrx TG mice, Picro Sirius Red staining was decreased compared to age-matched WT mice (19–22 months old, *n* = 7 each, *p* < 0.05) (Figure 1F,G). In addition, quantitative PCR showed a significantly lower expression of the fibrotic marker Acta2 in the liver of mice expressing hGlrx (Figure 1H). Our data indicate that an increase in Glrx levels attenuates age-induced fibrosis in the liver.

### 3.2. Modulation of Glrx Levels Controls Fibrosis and Apoptosis in the Aged Liver

**Figure 2:**
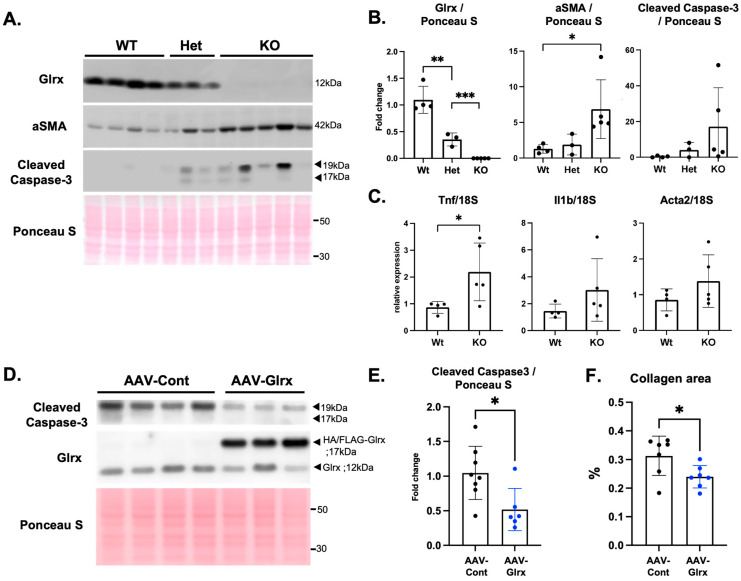
Glrx controls apoptosis and age-induced fibrosis in the liver. (**A**)**.** Protein expression of markers of fibrosis (αSMA) and apoptosis (cleaved caspase-3) in the liver of middle-aged WT, Glrx Het (Glrx^+/−^), and Glrx KO (Glrx^−/−^) mice (9–11 months old, males). (**B**). Semi-quantitative analysis of immunoblot bands normalized to Ponceau S intensities in each sample. (**C**)**.** Gene expression was measured by qPCR of the livers of middle-aged WT and Glrx KO mice (*n* = 3–5). Tnf: tumor necrosis factor, alpha; Il1b: interleukin1 beta; Acta2: alpha-actin 2. (**D**). Cleaved caspase-3 expression in the liver of aged mice with hepatocyte-specific overexpression of Glrx. Liver samples were prepared from 22-month-old mice three months after injection of the vector. Exogenous HA/FLAG-Glrx protein expression detected in the liver after AAV-Hep-Glrx injection is shown. (**E**). Semi-quantitative analysis of cleaved caspase-3 expression. (**F**). Collagen-stained area (%) in the liver after AAV-Hep-control or AAV-Hep-Glrx injection (*n* = 7, males and females, 22 months old). Blue dots on the graph indicate mice treated with AAV-Hep-Glrx. * *p* < 0.05, ** *p* < 0.01. *** *p* < 0.001. Error bars indicate standard error.

To further examine the role of endogenous Glrx in age-induced collagen accumulation in the liver, we analyzed middle-aged Glrx-deficient mice. Since Glrx knockout male mice spontaneously develop fatty liver with regular chow [22], we anticipated that lipid accumulation would cause damage to hepatocytes in the liver of Glrx knockout animals. Therefore, we examined markers of fibrosis and apoptosis in the liver of middle-aged (9- to 11-month-old) WT, Glrx^+/−^ (Het), and Glrx^−/−^ (KO) male mice. Protein levels of αSMA (Acta2) were increased in a dose-dependent manner by Glrx deficiency with a trend of increased cleaved caspase-3 (Figure 2A,B). In addition, the transcription of Tnf was also significantly higher in Glrx KO liver (Figure 2C), suggesting that Glrx has anti-inflammatory effects.

We hypothesized that higher levels of Glrx in hepatocytes could prevent apoptosis, resulting in reduced activation of stellate cells and fibrosis. To test this hypothesis, we injected AAVs carrying a hepatocyte-specific Glrx overexpression construct (AAV-Hep-Glrx) into aged (18- to 19-month-old males and females) WT mice to evaluate whether Glrx overexpression in hepatocytes protects against apoptosis and fibrosis in the aged liver. Three months after AAV administration, we confirmed that AAV-Hep-Glrx injection-induced expression of HA/FLAG-tagged Glrx and attenuated expression of cleaved caspase-3 in the liver (Figure 2D,E). In addition, mice treated with AAV-Hep-Glrx showed significantly less collagen deposition (Figure 2F). Taken together, the data demonstrated that AAV-mediated Glrx upregulation in hepatocytes can suppress mild fibrotic processes in the aged liver.

### 3.3. Modulation of Glrx Levels Controls Apoptosis in Hepatocytes in Vitro

**Figure 3:**
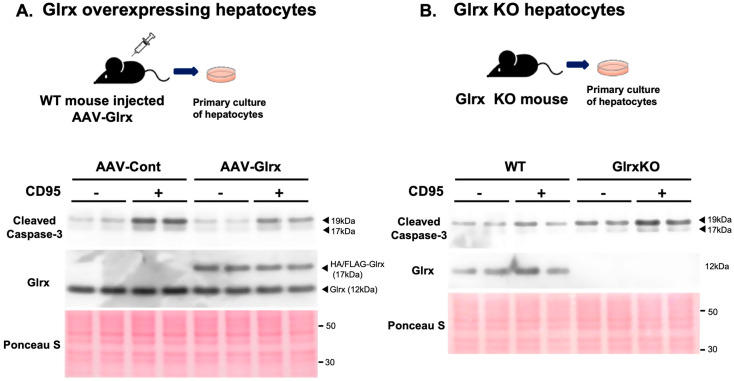
Glrx overexpression attenuates, and Glrx deficiency enhances apoptosis in mouse primary hepatocytes. (**A**). Three months after injection of adeno-associated virus (AAV) control vector or AAV containing a hepatocyte-specific Glrx overexpression construct, hepatocytes were isolated and treated with agonistic anti-CD95 antibody (1 µg/mL) for 24 h to induce cleaved caspase-3 as an apoptotic response. Overexpressed HA/FLAG-Glrx protein is detected at 17 kDa. (**B**). Glrx-deficient hepatocytes isolated from Glrx KO mice showed increased levels of cleaved caspse-3 following anti-CD95 treatment compared to those isolated from WT mice. These experiments were repeated more than three times and showed similar results.

To directly evaluate the anti-apoptotic role of Glrx in hepatocytes, we used primary hepatocytes treated with an agonistic antibody to FAS receptor (anti-CD95). Anti-CD95 antibody induces apoptosis through the activation of FAS signaling pathway. To see the effect of Glrx overexpression in hepatocytes, we injected AAV-Hep-Glrx or AAV-Hep-control vectors into WT mice, and isolated primary hepatocytes at 2 months post-injection. Then, we induced apoptosis in cultured cells by stimulation with an anti-CD95 (1 µg/mL, 24 h). CD95-induced expression of cleaved caspase-3 was attenuated in Glrx-overexpressing hepatocytes compared with the control AAV vector (Figure 3A). Conversely, when we induced apoptosis in primary hepatocytes obtained from 9-month-old WT and Glrx KO mice fed regular chow, the expression of cleaved caspases-3 was elevated in Glrx KO mice compared to WT mice (Figure 3B). Taken together with the data obtained in vivo, these in vitro experiments indicate that Glrx may suppress age-induced liver fibrosis by inhibiting apoptosis in hepatocytes.

### 3.4. Overexpression of Glrx Attenuates Liver Fibrosis in Diet-Induced NASH

**Figure 4:**
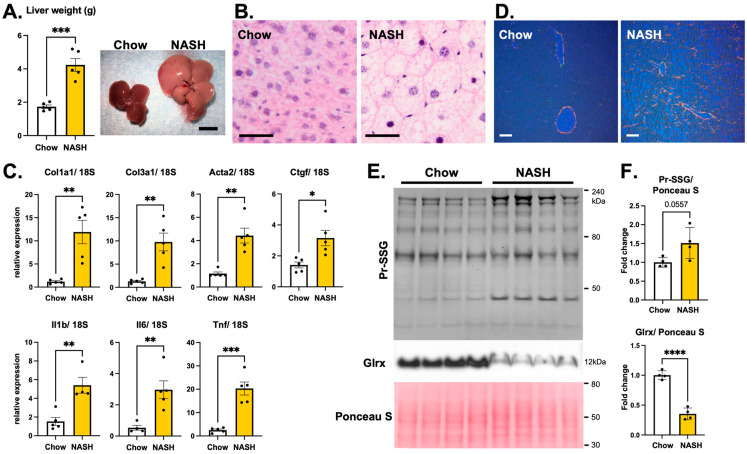
NASH diet induces fatty liver and fibrosis, inflammation, lower Glrx expression, and protein *S*-glutathionylation in the liver. (**A**). Liver weight of male C57BL/6J mice after 16 weeks on the NASH diet. The photo shows representative fatty liver induced by the NASH diet. (**B**). Representative image of H&E staining. Ballooning hepatocytes are induced by NASH diet. Scale bar: 20 um. (**C**). Quantitative PCR for markers of fibrosis and inflammation using livers of mice fed chow or NASH diet, normalized to 18S expression (*n* = 5). Col1a1: collagen type I, alpha 1; Col3a1: collagen type III, alpha 1; Acta2: alpha-actin 2; Ctgf: connective tissue growth factor; Il1b: interleukin–1 beta; Il6: interleukin–6; Tnf: tumor necrosis factor-alpha. (**D**). Representative Picro Sirius Red staining of liver from each group. Scale bar: 50 um. (**E**). Immunoblots show S-glutathionylated protein and Glrx protein in the liver. Ponceau S staining shows equal loading of proteins on the blot. (**F**). Semi-quantitative analysis of Pr-SSG and Glrx expression. * *p* < 0.05, ** *p* < 0.01, *** *p* < 0.001, **** *p* < 0.0001. Error bars indicate standard error.

We then examined the effect of Glrx overexpression on liver fibrosis in a diet-induced NASH model. Wild-type male mice (10-week-old) that are fed a high-fat high fructose diet (NASH diet) become obese and develop fatty liver with fibrosis and higher serum levels of AST and ALT at 15–16 weeks (about four months) (Figure 4A, Figure S1). H&E staining shows the ballooning of hepatocytes in the liver of NASH diet fed mice (Figure 4B). The transcription of fibrotic and inflammatory markers is elevated in the livers of animals fed a NASH diet compared to those fed regular chow (Figure 4C), as is collagen deposition (Figure 4D). Interestingly, Glrx protein levels are decreased in the livers of animals fed a NASH diet, with a concomitant increase in Pr-SSG levels (Figure 4E,F).

**Figure 5:**
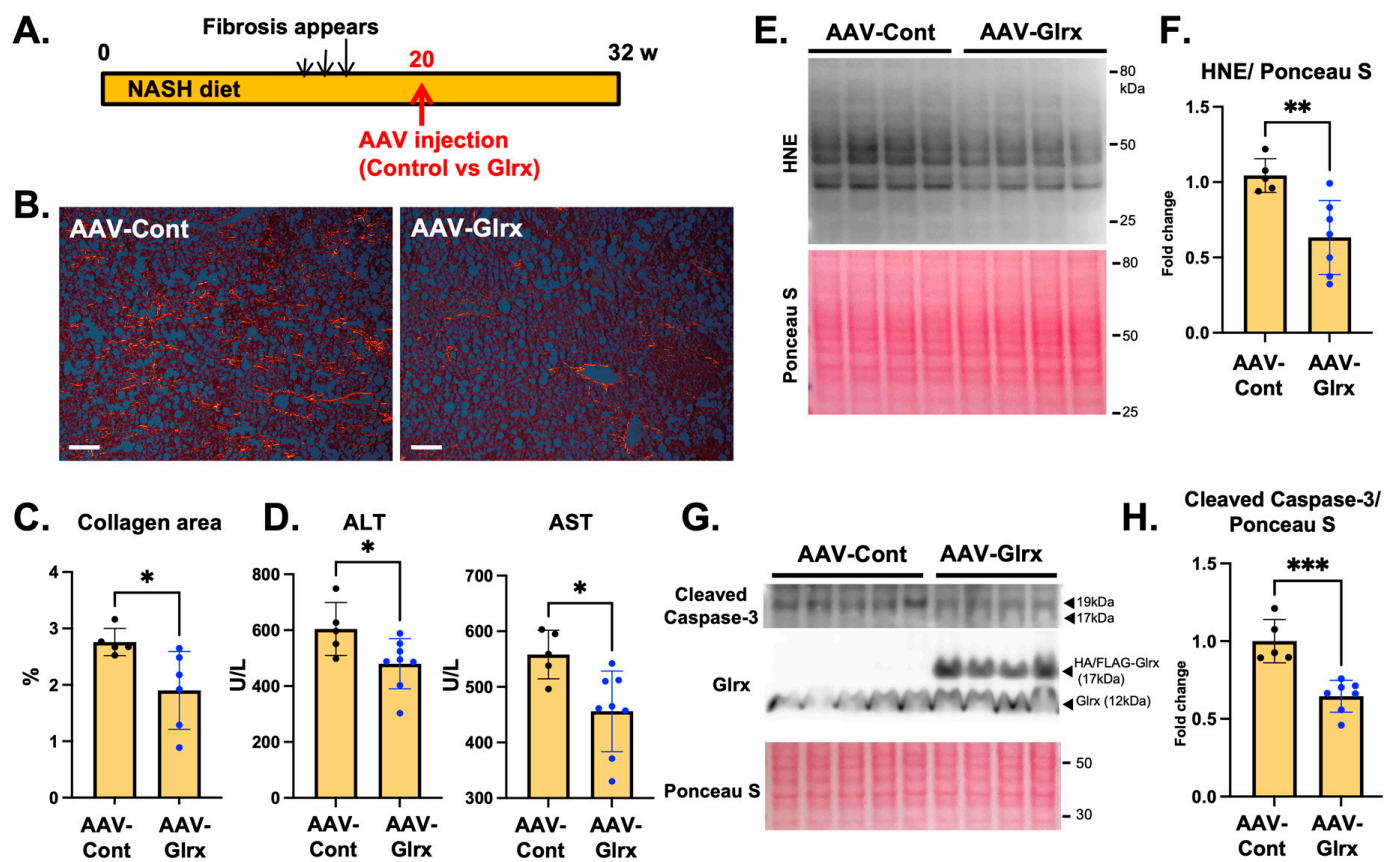
AAV-Hep-Glrx attenuates NASH-diet-induced fibrosis and improves liver function. (**A**). The experimental design shows time course. NASH with fibrosis appeared at 14–16 weeks of the NASH diet, and AAV-Hep-Glrx or AAV-Hep-control vector was injected at 20 weeks. The NASH diet was continued until euthanasia at 32 weeks. (**B**). Collagen deposition in the liver by Picro Sirius Red staining. Representative photos and quantitative assessment (% area) are shown (*n* = 5–6). (**C**). Semi-quantitative analysis of collagen staining area (%). (**D**). Serum liver enzyme levels. ALT: alanine aminotransferase, AST: aspartate aminotransferase (*n* = 5–8). (**E**). Immunoblot showing HNE (4-hydroxynonenal) levels. (**F**). Semi-quantitative assessment of HNE levels in the liver (*n* = 5–7). (**G**). Immunoblot showing cleaved caspase-3 expression in NASH liver treated with AAV-Hep-Glrx or AAV-Hep-control. (**H**). Semi-quantitative analysis of cleaved caspase-3 bands normalized to Ponceau S intensities. Blue dots on the graph indicate mice treated with AAV-Hep-Glrx. * *p* < 0.05, ** *p* < 0.01,*** *p* < 0.001. Error bars indicate standard errors.

To determine whether increased expression of Glrx could attenuate these effects, we injected AAV-Hep-Glrx or AAV-Hep-control vectors after 20 weeks of the NASH diet, when fibrosis had already appeared and continued the diet for another 12 weeks (Figure 5A). AAV-Hep-Glrx administration significantly decreased collagen accumulation in the liver and the serum levels of AST and ALT compared to AAV-Hep-control injection (Figure 5B–D). A lipid peroxidation marker, 4-hydroxynonenal (HNE) (Figure 5E,F), and cleaved caspase-3 expression (Figure 5G,H) were also decreased in the liver of AAV-Hep-Glrx-treated animals, indicating that hepatocyte damage is attenuated by Glrx overexpression. The cholesterol and triglyceride levels in the liver were not significantly lowered by AAV-Hep-Glrx (Figure S2). Therefore, although the administration of AAV-Hep-Glrx does not alter steatosis, this treatment can reduce NASH diet-induced hepatic apoptosis and fibrosis and improve liver function.

### 3.5. AAV-Glrx Provides Anti-Fibrotic Benefits to Dietary Modification in NASH

**Figure 6:**
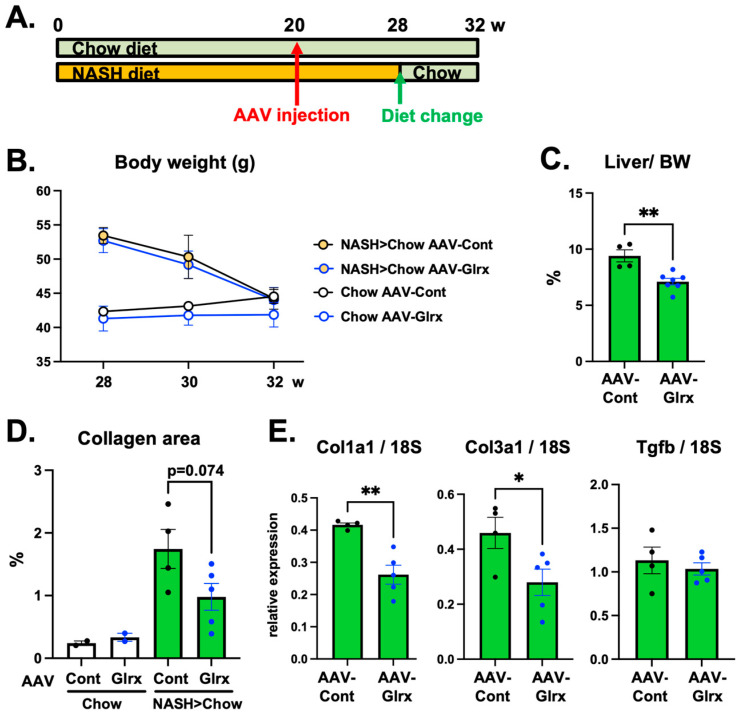
AAV-Hep-Glrx has a supplemental anti-fibrotic effect on diet modification. (**A**). Experimental design. AAV-Hep-Glrx or AAV-Hep-control was injected into mice fed chow or NASH diet (20 weeks). After 28 weeks NASH diet, a cohort was switched to regular chow for four more weeks. (**B**). Time course of bodyweight of each group after changing to chow diet. (**C**). Liver weight as a percentage of bodyweight showing the effect of AAV-Hep-Glrx. (**D**). Collagen staining area (%) by Picro Sirius Red staining. (**E**). Gene expression of fibrotic markers of the liver after dietary modification (*n* = 4–5). Blue dots on the graph indicate mice treated with AAV-Hep-Glrx. * *p* < 0.05, ** *p* < 0.01. Error bars indicate standard error.

Clinically, dietary therapy to reduce fat and calorie intake is a primary intervention for NAFLD/NASH. To model this dietary modification, we changed the NASH diet to regular chow for a month for a cohort of mice and found that the liver size and bodyweight of these animals returned close to those of animals fed only regular chow (Figure S3A–C). Furthermore, to examine whether Glrx has a complemental anti-fibrotic effect whereby dietary changes improve obesity and fatty liver, we injected AAVs at 20 weeks and then changed diet at 28 weeks (Figure 6A). In these animals, the bodyweight of both AAV-Hep-control and AAV-Hep-Glrx groups dropped similarly after changing the diet (Figure 6B). However, AAV-Hep-Glrx treatment further reduced liver size (Figure 6C) and also decreased collagen accumulation and the transcription of collagen genes (Col1a1, Col3a1) in the liver compared to AAV-Hep-control (Figure 6D,E). Our data, therefore, indicate that increased Glrx expression leads to an anti-fibrotic effect in the liver complementarily to diet therapy.

### 3.6. AAV-Glrx Downregulates Immune-Related Pathways in NASH Liver

**Figure 7:**
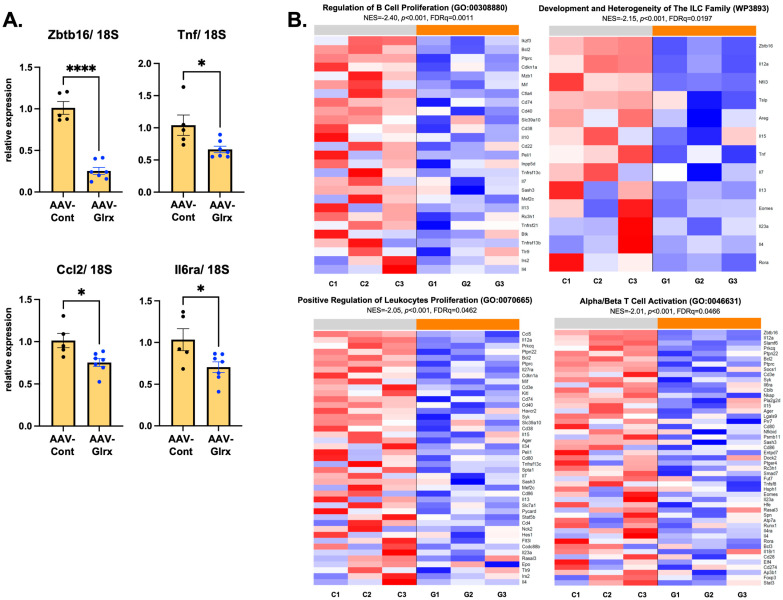
AAV-Hep-Glrx effects on transcriptome on diet-induced NASH liver. (**A**). Quantitative qPCR of livers of NASH-diet-fed mice treated with AAV-Hep-Glrx or AAV-Hep-control. Zbtb16: zinc finger and BTB domain-containing 16, Tnf: tumor necrosis factor-alpha, Ccl2: CC motif chemokine ligand 2, Il6ra: IL–6 receptor alpha. Blue dots on the graph indicate mice treated with AAV-Hep-Glrx. * *p* < 0.05, **** *p* < 0.0001. Error bars indicate standard error. (**B**). Heatmaps of the leading edges of immune-related gene sets with significant coordinate downregulation (FDR *q* < 0.25) in response to AAV-Hep-Glrx administration. Rows correspond to genes, and columns correspond to samples (C1–C3: mice treated with AAV-Hep-control; G1–G3: mice treated with AAV-Hep-Glrx). Blue and red indicate expression values at least 2 standard deviations below or above the mean (white) of each row, respectively. Genes are arranged in descending order from top to bottom by the magnitude of the moderated *t* statistic.

We have demonstrated that Glrx prevents apoptosis in hepatocytes, which prevents stellate cell activation and fibrosis in the liver. Furthermore, hepatocyte-specific overexpression of Glrx shows a similar anti-fibrotic effect in animals fed a NASH diet. To elucidate the target genes and pathways by which hepatic Glrx may mediate this effect, we used whole-genome gene expression microarrays to profile livers from animals on a NASH diet transfected with AAV-Hep-control AAV-Hep-Glrx vectors using samples from Figure 5. Microarrays showed that only one gene, Zbtb16, was significantly regulated by AAV-Hep-Glrx after correction for multiple hypothesis testing (4.3-fold decrease in AAV-Glrx, *p* = 3.3 × 10^−6^, FDR *q* = 0.09), although the expression of several inflammatory markers also trended downward in AAV-Hep-Glrx-treated animals (Tnf: 1.1-fold, *p* = 0.093; Ccl2: 1.2-fold, *p* = 0.33; Il6ra: 1.3-fold, *p* = 0.060) (Table S1). Quantitative PCR confirmed that the expression of these genes was significantly reduced in AAV-Hep-Glrx treated liver (Figure 7A). We then used Gene Set Enrichment Analysis (GSEA) to identify pathways that were coordinately regulated in response to AAV-Hep-Glrx administration. This analysis showed that 226 gene sets were significantly coordinately downregulated (FDR *q* < 0.25) by AAV-Hep-Glrx compared to AAV-Hep-control (Table S2), including those related to the regulation of various immune cells (including B-cells, T-cells, innate lymphoid cells, and leukocytes) (Figure 7B). These data suggest that the upregulation of Glrx in hepatocytes may negatively regulate hepatic immune cells in the setting of diet-induced NASH.

## 4. Discussion

In this study, we have shown that Glrx deficiency elevates markers of fibrosis and apoptosis both in vivo and in vitro, whereas augmenting Glrx expression protects hepatocytes from cell death and decreases collagen levels in the aged liver and diet-induced NASH liver. Age-induced fibrosis is mild and may not affect liver function per se but promotes fibrosis and NASH progression in the context of a high-fat diet [18,19]. We, therefore, anticipated that administration of Glrx could protect the liver from pathological fibrosis in diet-induced NASH. In this study, we fed C57BL/6J mice a NASH diet containing high fructose and palmitate, which induced steatohepatitis with fibrosis in 15–16 weeks. We aimed to determine if Glrx in hepatocytes may influence stellate cells and immune cells. Therefore, we treated these mice with a hepatocyte-specific AAV-Hep-Glrx expression vector after 20 weeks of the NASH diet and continued the diet for another 12 weeks before examining the liver. Interestingly, the livers of mice fed a NASH diet express lower levels of Glrx and have higher Pr-SSG levels than the livers of chow-fed mice. This is similar to the pattern of Glrx expression reported in the fibrotic lung: Glrx expression is downregulated in the lungs of idiopathic pulmonary fibrosis patients and Glrx overexpression protects against experimental lung fibrosis [26]. The impact of a NASH diet appears to override the metabolic effects of Glrx because AAV-Hep-Glrx administration did not lead to a significant decrease in steatosis. However, AAV-Hep-Glrx attenuated an apoptotic marker and collagen accumulation and improved ALT and AST levels in the liver.

Glrx inhibits Fas signaling, protects lung epithelial cells from apoptosis, and attenuates experimental lung fibrosis [27], and can mediate anti-apoptotic effects by activating NFκB [28,38,39] or SirT1 [25,40] via the reversal of *S*-glutathionylation. Accordingly, our data show that Glrx regulates Fas-induced apoptosis in hepatocytes and may, therefore, suppress fibrotic processes in the liver through anti-apoptotic regulation.

High-fat-diet-induced lipotoxicity causes hepatocyte damage and cell death, which in turn activates immune cells and stellate cells, leading to fibrosis and to the progression of NAFLD and NASH [41]. Apoptotic markers and Fas expression were increased in the liver of NASH patients [42,43], and a pharmacological caspase inhibitor reduced fibrosis in a NASH mouse model [44]. We detected cleaved caspase-3 as an apoptotic marker in NASH livers at 32 weeks, which was attenuated by AAV-Hep-Glrx treatment. In addition, the lower ALT and AST values and HNE levels in AAV-Hep-Glrx-treated animals indicate that Glrx protects hepatocytes against cell death caused by oxidative stress and lipotoxicity. Our previous report showed that Glrx overexpression reversed lipid accumulation and metabolic gene dysregulation in Glrx KO mice fed regular chow, suggesting that Glrx may control lipid metabolism [22]. However, Glrx overexpression did not improve lipid metabolism in our NASH model (Figure S2). Presumably, NASH diet-induced lipotoxicity may overwhelm the metabolic effect of Glrx overexpression. We found that liver/bodyweight (%) decreased by AAV-Hep-Glrx after the diet changed to regular chow, demonstrating the effects of Glrx on lipid metabolism with a regular diet. In addition, it could be possible that starting the AAV-Hep-Glrx treatment earlier may have improved diet-induced steatosis. Unexpectedly, we found that upregulation of Glrx in hepatocytes suppressed the immune cells pathways, including proliferation, differentiation, and activation of B- and T-lymphocytes and leukocytes, chemokine production, and chronic inflammatory responses. These results suggest that Glrx decreases liver fibrosis in NASH by its anti-apoptotic and anti-inflammatory effects.

Intriguingly, AAV-Hep-Glrx treatment also significantly downregulated transcription of the gene Zbtb16 (zinc finger and BTB domain-containing 16), which encodes the transcription factor PLZF (promyelocytic leukemia zinc finger) that controls lymphocyte differentiation [45] and metabolic syndrome [46]. Reactive oxygen species production is also decreased in natural killer T (NKT) cells in the liver of PLZF^+/−^ mice [47], suggesting that Zbtb16 may negatively regulate antioxidant signaling. A subset of NKT cells contribute to diet-induced steatosis and fibrosis [48,49], and B-cell involvement in NAFLD/NASH has been shown in human and mouse studies [50]. Therefore, decreased expression of Zbtb16 and suppression of the proliferation and activation of immune cells may explain the protective mechanism of Glrx in NASH.

Hepatocytes produce innate immunity proteins and pro-inflammatory cytokines under stress conditions. One of the liver-enriched transcription factors that may secrete immune mediators in an acute response is CCAAT/enhancer-binding protein (C/EBP) β [51,52]. C/EBPβ is stabilized and activated by *S*-glutathionylation in adipocytes [53] and may, therefore, be one of the targets by which Glrx decreases inflammatory mediators in NASH. However, further investigation is needed to elucidate how Glrx expression in hepatocytes regulates Zbtb16 expression and immune cells, particularly lymphocytes, in the NASH liver. Analyzing secretory molecules from Glrx-overexpressing hepatocytes will be interesting to identify mediators that control stellate cells and immune cells. In addition, hepatic Zbtb16 mRNA expression is augmented by a high-fat, high-cholesterol diet [54], but the role of Zbtb16 in hepatocytes is unknown and is a potential research topic for the future.

## 5. Conclusions

We have demonstrated that the upregulation of Glrx can reverse age-induced apoptosis and fibrosis in the liver and ameliorate fibrosis in diet-induced NASH. AAV-mediated hepatocyte-specific Glrx expression in vivo decreased collagen accumulation, improved hepatic function, and suppressed immune cell activation and inflammation in NASH livers. The data indicate crosstalk between hepatocytes and other cells to control fibrotic processes in the liver, showing that Glrx has the potential to be a powerful anti-fibrotic therapeutic molecule.

## Figures and Tables

**Table 1:** Primer sequences used for quantitative PCR.

Gene	Gene Accession	Forward Primer	Reverse Primer
Ccl2	NM_011333	5′-TTAAAAACCTGGATCGGAACCAA-3′	5′-GCATTAGCTTCAGATTTACGGGT-3′
Col1a1	NM_007742	5′-CCCGAACCCCAAGGAAAAGA-3′	5′-CGTACTCGAACGGGAATCCA-3′
Col3a1	NM_009930	5′-CAGAGGAGAAACTGGCCCTG-3′	5′-TTGTCACCTCGTGGACCTTG-3′
Ctgf	NM_010217	5′-CTAGCTGCCTACCGACTGGA-3′	5′-GTAACTCGGGTGGAGATGCC-3′
Il1b	NM_008361	5′-GCAACTGTTCCTGAACTCAACT-3′	5′-ATCTTTTGGGGTCCGTCAACT-3′
Il4	NM_021283	5′-GGTCTCAACCCCCAGCTAGT-3′	5′-GCCGATGATCTCTCTCAAGTGAT-3′
Il6ra	NM_010559	5′-CCTGAGACTCAAGCAGAAATGG-3′	5′-AGAAGGAAGGTCGGCTTCAGT-3′
Tgfb1	NM_011577	5′-GCTGAACCAAGGAGACGGAA-3′	5′-ATGTCATGGATGGTGCCCAG-3′
Tnf	NM_013693	5′-ATGGCCTCCCTCTCATCAGT-3′	5′-CTTGGTGGTTTGCTACGACG-3′
Zbtb16	NM_001033324	5′-CTGGGACTTTGTGCGATGTG-3′	5′-CGGTGGAAGAGGATCTCAAACA-3′
18S RNA	NR_003278.3	5′-CCGCCGCCATGTCTCTAGT-3′	5′-GCCCATCGATGTTGGTGTTG-3′

## Data Availability

The data presented in this study are openly available in the Gene Expression Omnibus (GEO) through Series accession number GSE198961 (https://www.ncbi.nlm.nih.gov/geo/query/acc.cgi?acc=GSE198961).

## Supplementary Materials

The following supporting information can be downloaded at: https://www.mdpi.com/article/10.3390/antiox11050867/s1, Figure S1: NASH diet effects on bodyweight and hepatic function; Figure S2: effects of AAV-Glrx on lipid levels in NASH liver; Figure S3: effects of diet changes on bodyweight and liver; Table S1: gene expression microarray profiling of liver from animals fed a NASH diet and treated with AAV-control or AAV-Glrx; Table S2: gene set enrichment analysis.
