# Supplementary material for: Administration of Glutaredoxin-1 Attenuates Liver Fibrosis Caused by Aging and Non-Alcoholic Steatohepatitis
Source: Antioxidants (Basel). 2022 Apr 28;11(5):867. doi: 10.3390/antiox11050867 (PMC9138033; doi:10.3390/antiox11050867)
Supplement: Supplementary file 1 [file antioxidants-11-00867-s001.zip › Supplemental Figures.pdf]

Figure S1. NASH diet induces obesity and hepatic dysfunction

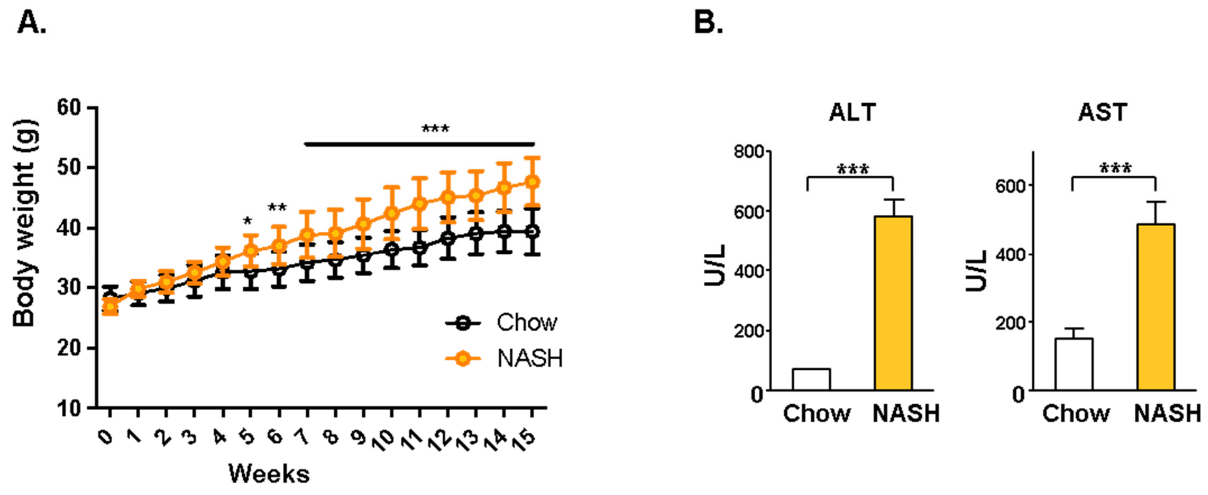

A. Body weight increase after feeding WT male mice (10 week-old) NASH diet or regular chow for 16 weeks. (n=5). **B.** Serum levels of ALT and AST after 16 weeks of diet. * p<0.05, ** p< 0.01, *** p<0.001. Error bars indicate standard error.

Figure S2. AAV-Glrx did not suppress lipid levels in NASH liver

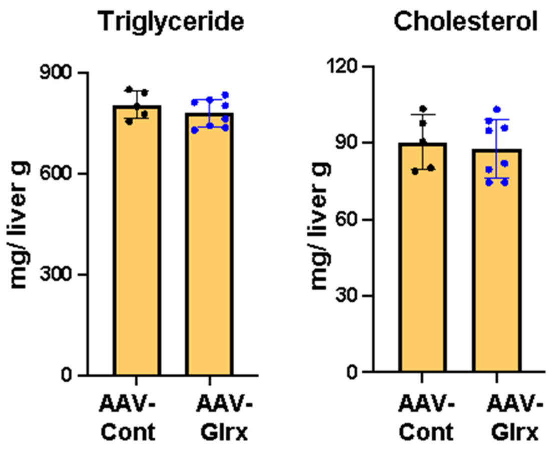

Triglyceride and cholesterol were measured in the liver of mice fed NASH diet for 32 weeks. AAV-Glrx treatment did not show the difference compared to AAV-Control treatment. (See Figure 5A)

Figure S3. **Effects of diet change in body weight and liver**

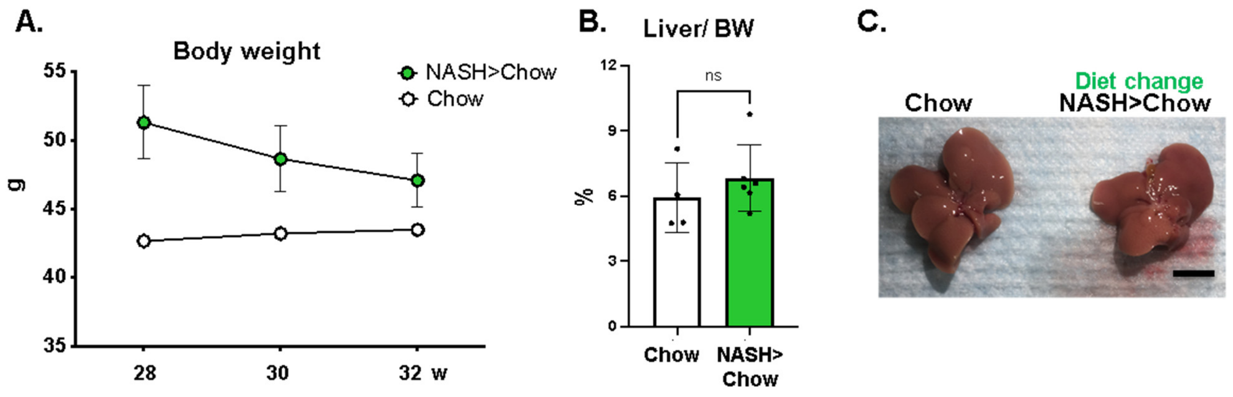

WT mice (10 week-old) were fed NASH diet for 28 weeks, changed to regular chow for 4 weeks, and then euthanized. **A.** Body weight after the diet change. Green circles indicate mice fed NASH diet first. White circles indicate mice fed only regular chow. **B**. Liver weight over body weight (%) (ns: no significance, n=5-6). **C**. Representative photo of a liver after diet change compared to chow-fed mouse liver.
